# Effect of Gold Nanoparticle on Structure and Fluidity of Lipid Membrane

**DOI:** 10.1371/journal.pone.0114152

**Published:** 2014-12-03

**Authors:** Anil R. Mhashal, Sudip Roy

**Affiliations:** Physical Chemistry Division, National Chemical Laboratory, Dr. Homi Bhabha Road, Pune, 411008, India; University of Akron, United States of America

## Abstract

This paper deals with the effect of different size gold nanoparticles on the fluidity of lipid membrane at different regions of the bilayer. To investigate this, we have considered significantly large bilayer leaflets and incorporated only one nanoparticle each time, which was subjected to all atomistic molecular dynamics simulations. We have observed that, lipid molecules located near to the gold nanoparticle interact directly with it, which results in deformation of lipid structure and slower dynamics of lipid molecules. However, lipid molecules far away from the interaction site of the nanoparticle get perturbed, which gives rise to increase in local ordering of the lipid domains and decrease in fluidity. The bilayer thickness and area per head group in this region also get altered. Similar trend, but with different magnitude is also observed when different size nanoparticle interact with the bilayer.

## Introduction

Lipids are the major component of cell membrane and the phospholipids are one of the abundant class of membrane lipids. The phospholipid membranes serve as a barrier and selectively allow molecules to the interior of the cell. As the cell is the central part of life, the understanding of the functionality of cell wall i.e. lipid bilayer membrane under the influence of foreign material is a major challenge in biology. Living bodies are always exposed to the nanoparticles (NP) of different size, which inevitably leads to experimentation to understand the risk and hazard associated with NPs. Cytotoxicity effects of NPs are well known and mostly depend on the size of NPs [1], [2]. Any foreign particles, e.g. NPs, polymers, etc. enter the cell membrane by two different ways, either by endocytosis or by diffusing through the membrane [2], [3], which is vastly dependent on the size of the particle. Computer simulations showed that NPs with small size (2–8 nm) get embedded into the bilayer and it is thermodynamically favorable [4]. On the other hand, hydrophobic and hydrophilic nature of the NPs also play significant role in the embedding process of NPs in the bilayer. Hydrophilic NPs generally get adsorbed and assembled at the bilayer-water interface whereas hydrophobic NPs get accumulated easily in the hydrophobic region of the bilayer, which facilitate higher loading of the NPs in the bilayer. However, the process of insertion of hydrophobic NP in the bilayer is difficult [5], [6]. One more important parameter, in case of penetration, is the charge on the surface of the NPs. Li and Gu [7] studied the adsorption of charged NPs by coarse grained molecular dynamics simulations. They reported that the electrostatic interaction between NP and bilayer facilitates adhesion of the charged NP to the membrane, which induces local transitions in fluid bilayers. The shape of the nanoparticle also plays a vital role, which directs the insertion mechanism. Yang et al. investigated the physical translocation of NPs with different shapes like spheres, ellipsoids, rods, discs and pushpin-like particles in the lipid bilayer [8]. The study also showed that the volume of the particle plays significant role in penetration process and rotation of particle in the interface complicates the process of insertion. Computer simulations of interaction of graphene [9] and fullerene [10] are also being investigated to understand the translocation process of these low-dimensional systems.

Gold nanoparticles (AuNP) are being used as drug delivery agent [11], medical diagnostics [12] and as therapeutic agent [13], [14]. AuNPs are successfully tested as gene delivery agent [15] and in cancer therapy [16]. Small sized hydrophobic AuNP (less than 2nm, coated with dodecanethiol for hydrobhobicity) can enrich hydrophobic areas of lipid bilayer by hydrophobic interactions. Korgel et al. [17] has explained how this enrichment of capped hydrophobic AuNP occurs in the bilayer. The hydrophobic nanoparticles can unzip the lipid bilayers and get loaded in the hydrophobic part of the membrane.

The loading of nanoparticles in bilayer can change in the phase diagram of a lipid bilayer. Bothum [18] showed experimentally that augmentation of decanethiol-capped AuNPs into liposomal membrane decreases the melting temperature at high concentration of nanoparticles. Similarly Sung-Sik Han et al. [19] also showed entrapped silver nanoparticles in 1,2-dipalmitoyl-sn-glycerol-3-phosphocholine (DPPC) liposome fluidifies the membrane, which might be because of interactions between the DPPC lipid molecules and silver nanoparticle.

To understand the interactions between nanoparticle and lipid molecules, molecular dynamics (MD) simulation of a coarse grained model of gold nanoparticle and model lipid membrane was performed by Zheng et al. [20]. They observed coarse grained nanoparticles of size 2.2 nm with different signs of the charges and densities of surface charge naturally adsorb to the model bilayer surface or penetrate into the bilayer.

However, all the experimental and theoretical studies mentioned above are mostly dealt with the local interactions of the nanoparticle with the bilayer molecules and thereby penetration mechanism of the particles in the bilayer. The missing link to all these investigations is the effect of the NPs on the lipid molecules, which are not directly interacting with the NPs.

In this present study, we aim to simulate neutral AuNPs of different size with lipid bilayer using all atomistic MD to address the effect of non-passivated NP on the lipid molecules (which are not directly interacting) for the first time to the best of our knowledge. In this work we intend to understand the effect non-passivated NP, however, most of the work on AuNP has been carried out with surface capping agents. These surface passivating agents then change the chemical nature of the AuNP. Merga et al. recently synthesized naked AuNP by reducing Au_2_O_3_ by molecular hydrogen [21]. Zopes et al. synthesized small size naked AuNP by hydrolysis of Gold complex [NMe_4_][Au(CF_3_)_2_] [22]. Caprile et al. studied the interaction of naked AuNP with L-cysteine using high resolution XPS [23]. Therefore interaction of non-passivated AuNP with lipid bilayer is an important aspect to address from molecular level simulation.

We have considered bilayer made of lipids with unsaturation in one of the aliphatic tail because of the higher fluidity in such assembled membrane. Therefore possibly, the change in magnitude of the fluidity of such membrane interacting with AuNP will be higher than bilayer composed of saturated lipid molecules. The fluidity in membrane is directly related to the structural arrangement of the lipid molecules. However, this structural arrangement of lipid may differ for adjacent to and far from the NP adsorption site (interaction site). Therefore, we have correlated fluidity in different parts of membrane (adjacent and far from the nanoparticle) to the ordering of the lipids. Apart from fluidity, we have also analyzed local structure of lipids near to AuNPs, the diffusivity of lipid molecules in presence of AuNP and densities of water around AuNP, which has deeply penetrated the bilayer.

### Computational Method

We have performed all atomistic MD simulations of 1-arachidoyl-2-oleoyl-sn-glycero3-phosphocholine (AOPC) with different size of AuNPs separately. To set up the simulations, we have used three consecutive processes, which includes a) self-assembly of lipid, b) construction of AuNP followed by its simulation in vacuum and water c) simulation of self-assembled lipid and AuNP systems.

### Self-assembly of lipid

Poger D. et al. [24] studied the lipid model with mixed acid phosphatidylcholine (PC) and developed parameter sets for various such lipid molecules. In the present study, we have used a united atom model of AOPC (the structure of AOPC is shown in Fig. S1) extracted from the parameter set for PC (force field parameters are given in Text S1). The lipid model system contains monounsaturated oleoyl (18 Carbon atoms in aliphatic chain, Fig. S1) with slightly longer saturated arachidoyl (20 Carbon atoms in aliphatic chain, Fig. S1). It is experimentally shown that lipid rafts with one saturated fatty acid chains are involved in signaling [25] and transport of molecules [26]. It is also found that raft phospholipids contain longer saturated fatty acid chains [27], [28] e.g., palmitic, stearic, and arachidic acid when compared to unsaturated fatty acids.

We have performed self-assembly simulation of AOPC lipids starting from 128 randomly distributed AOPC molecules in water. We have used SPC water model [29] for all the simulations. The simulations were performed using the GROMACS 4.5.1 [30]–[32] MD code. Isobaric-isothermal (NPT) ensemble with periodic boundary conditions (PBC) was used for simulations. Phase transition temperature of AOPC is 284.9K [33]. Therefore, we have used 300K and 1 bar pressure for self-assembly simulation. Temperature coupling was used with v-rescale algorithm [34] with a coupling constant of 0.1 ps. The isotropic pressure coupling was applied by using Berendsen barostat [35] only for the self-assembly simulation. The pressure coupling time constant was 2.0 ps in order to maintain a constant pressure of 1.0 bar. Bonds were constrained with LINCS algorithm. The Lennard-Jones interactions were taken care with cut-off of 1.4 nm. For electrostatic interactions Particle-Mesh Ewald (PME) [36] method was used with a cut-off of 1.4 nm. After 50 ns MD simulation, we have obtained the self-assembled bilayer of AOPC.

### Construction of AuNP

The initial structures of AuNPs were constructed from *fcc* lattice of Gold. In crystalline lattice the center of the nanoparticle was set on a gold atom, then a radius 1 nm, 1.75 nm and 2.5 nm was considered. Gold atoms fall into this radius were taken as part of nanoparticles of different sizes of diameter 2 nm (2 nm_AuNP), 3.5 nm (3.5 nm_AuNP) and 5 nm (5 nm_AuNP). The initial structures of AuNP are shown in Fig. S2. These AuNPs were further energy optimized and subjected to MD simulation in vacuum and in water for 5 ns to check the stability and structural relaxation. Gold atoms in the nanoparticle interact only via Lennard-Jones (LJ) potential. These LJ parameters are obtained from the paper of Heinz et al.[37]. The parameters suggest that the AuNPs are hydrophilic is nature. The final snapshots after 5 ns of MD run of nanoparticle in water are shown in Fig. S2. Distance distribution plots for gold atoms in a vacuum and water from last 1 ns of MD trajectories are shown in Fig. S3. The structures of the nanoparticle were retained intact and stable throughout the simulations (see Fig. S4a). In Fig. S4b, the radial distribution function (RDF) between Gold and Oxygen atom of water shows the hydrophilic nature of AuNP. As the head-group of lipid membrane is hydrophilic, it is expected that hydrophilic AuNP will interact favorably with the lipid head-groups. Note that in the present study, gold nanoparticles do not possess any charges on the surface.

### Lipid-nanoparticle system

We aim to study the interaction of AuNP with AOPC lipid bilayer and its effects on the bilayer properties, therefore, we have assembled the nanoparticles of different sizes on the surface of the bilayer and constructed following three systems.

**Figure 1:**
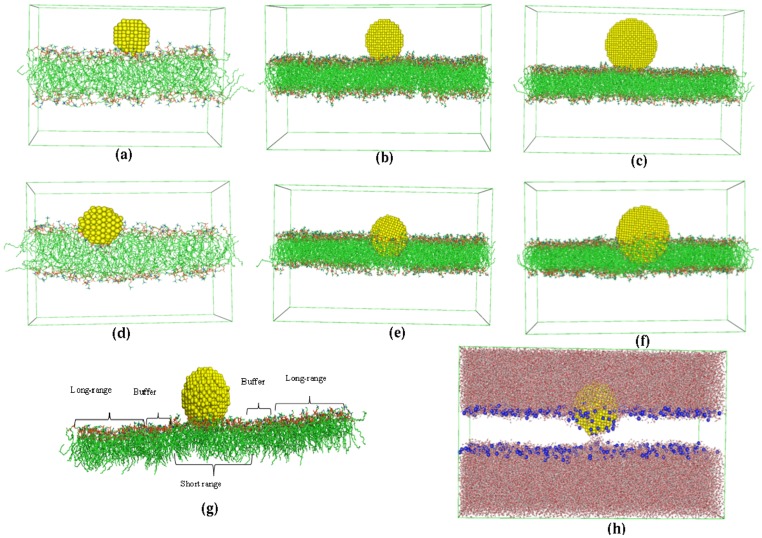
Initial and final snapshots after 100 ns MD simulation of three systems. (a, d) 2 nm_AuNP (b, e) 3.5 nm_AuNP and (c, f) 5 nm_AuNP. (g) Schematic representation of the short range (SR), long range (LR) and buffer region. (h) snapshot of 3.5 nm_AuNP system with explicitly represented head-group Nitrogen atoms in blue color.

The smallest sized nanoparticle 2 nm_AuNP was placed initially on the self-assembled AOPC bilayer of 128 molecules (∼64 molecules on each leaflet). For the second and third system with 3.5 nm_AuNP and 5 nm_AuNP particles, we have replicated the above bilayer in x and y direction (in each bilayer direction). Before placing the AuNP, the replicated bilayer was subjected to equilibration run for 10 ns. Thereafter, the nanoparticles were placed on the surface of the leaflet of bilayer (in water phase) in such a way that surface atoms of both nanoparticle and lipids fall in the range of van der Waals cut-off. The non-bonded interactions between the AuNP atoms and bilayer atoms are calculated by using the combination rule of geometric mean for all C^(6)^ and C^(12)^ parameters. To compare the effects of nanoparticle on bilayer, we have also simulated AOPC bilayer of 512 lipid molecules without a nanoparticle for 100 ns and termed as reference lipid bilayer. The snapshots of self-assembled bilayer with 128 lipid molecules and replicated bilayer with 512 lipid molecules without AuNP are showed in Fig. S5. All the three systems described above with AuNP in their initial positions are depicted in Fig. 1a, b, and c and the number of atoms of each component, i.e., Gold, AOPC and water, and equilibrated simulation box size are given in Table 1. These systems were solvated with SPC water [38] and simulated for 100 ns with semi-isotropic pressure coupling using Berendsen barostat [35] with separate coupling to xy-plane and z-direction (the bilayer normal). The remaining parameters for MD were kept same as used in self-assembly simulation. The simulation trajectories were written after each 5 ps and used for analysis. In this work we have analyzed the effects of nanoparticle on membrane in two different regions; near to the adhesion (interacting) site and away from the interacting site. Therefore, we have divided the membrane based on the distance from nanoparticle to the lipid molecules in interacting leaflet only. As we have used short range cut-off of 1.4 nm, we have considered the interacting short range (SR) lipid molecules (coordinates of head group Nitrogen atoms) which fall into the distance of 1.5 nm + radius of nanoparticle (r). E.g., for 2 nm_AuNP the interacting lipid molecules are those, which fall into the distance of 2.5 nm from the center of mass (CoM) of AuNP. To understand the indirect effect of nanoparticle on bilayer; we have considered a region, which is away from interacting short ranged lipid molecules as defined above. As SR lipids can diffuse with time, therefore to get rid of hard boundary between SR molecules and rest of lipid molecules, we have defined a region as a buffer. The lipid molecules fall in between distance greater than (r+1.5 nm) and less than (r+ 1.5 nm + 0.5 nm) are taken as buffer region lipid molecules and are not considered for any analysis. Rest of the lipid molecules in the same leaflet, which are at a distance greater, than (r+ 1.5 nm + 0.5 nm) are not directly interacting with AuNP, are termed as long range (LR) lipid molecules (Fig. 1g). As lipid molecules (and AuNP) are dynamic in nature we have updated these boundaries (between SR – buffer and buffer – LR region) every 10 ns and tagged the lipid molecules of these respective regions for further analysis. To calculate the properties of long range molecules we have considered the sufficiently large system with 512 lipid molecules which gives a total of 301640 atoms (for details see Table 1) including water and nanoparticle. For all analysis we have used lipid molecules from interacting (with AuNP) leaflet. Schematically we have shown SR, LR and buffer molecules in the bilayer in Fig. 1g, and the number of the lipids falls into respective regions is showed in Table S1. All the analysis programs are discussed along with the results and discussions.

**Table 1:** Details of the bilayer AuNP systems.

System	No of gold atoms	AOPC molecules	No. of AOPC united atoms	No. water molecules	System size in atoms	Simulation box size (nm)		
						X	Y	Z
**Reference System**	0	512	28672	48949	175519	22.06	11.13	9.09
**2 nm_AuNP**	249	128	7168	11008	40441	11.01	5.55	8.52
**3.5 nm_AuNP**	1289	512	28672	76619	259818	22.32	11.16	12.5
**5 nm_AuNP**	3926	512	28672	89681	301640	22.38	11.19	14.3

## Results and Discussions

### Structural properties of interacting molecules with AuNP

**Figure 2:**
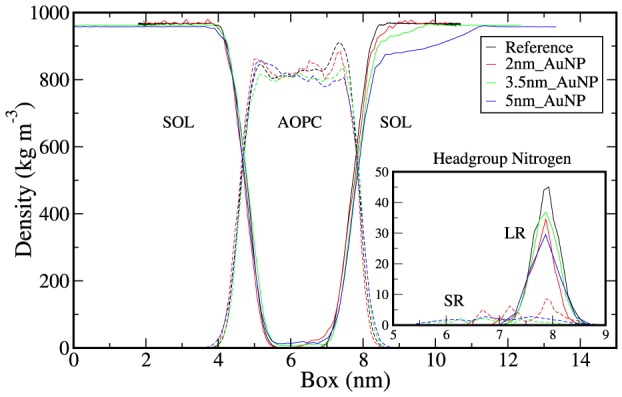
Partial densities of water and AOPC. Dotted lines represents lipid and solid lines represents the water partial densities. Black, red, blue and green lines represent reference, 2 nm_AuNP, 3.5 nm_AuNP and 5 nm_AuNP systems respectively. Figure Inset represents partial densities of head–group Nitrogen atoms of SR and LR lipid of interacting lipid monolayer where dotted lines shows SR lipids and solid lines shows LR lipids.

**Figure 3:**
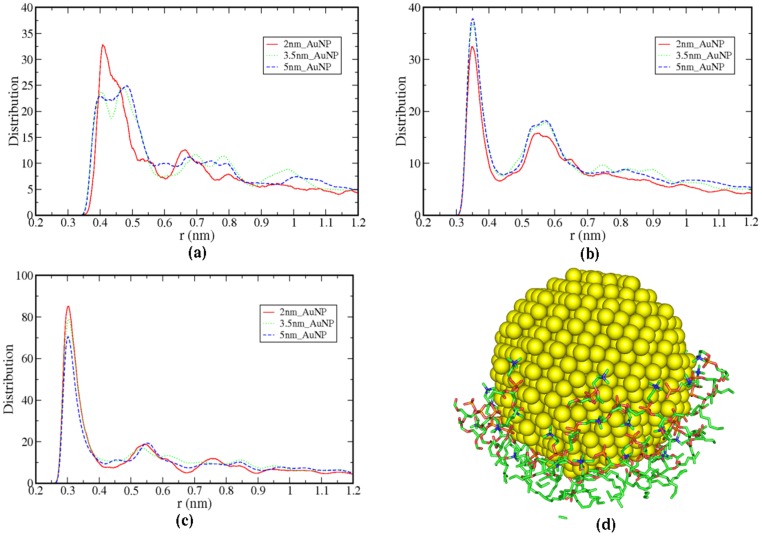
Distance distribution between surface Gold atoms of AuNP and (a) head-group Nitrogen, (b) NMe group's C atoms and (c) non-ester Oxygens of Phosphate group respectively. d) Snapshot of lipid headgroup interaction with AuNP.

Four systems, including the reference system (without AuNP) are structurally characterized to see the position of the nanoparticle as a function of time. The snapshots of the systems as a function of time (0 ns, 25 ns, 50 ns, 75 ns and 100 ns) are given in Fig. S6. From the snapshots at 100 ns in Fig. 1d, e, f (intermediate snapshots at Fig. S6) it is evident that the AuNP of different sizes gets adsorbed on the bilayer surface and penetrates deeper in the bilayer along with some of the head groups of the lipid molecules (Fig. 1h). From partial density plots (Fig. 2), it is clear that the head group nitrogen atoms penetrate deep into the bilayer along with AuNP. In Fig. 2 (inset) nitrogen densities of interacting SR and LR lipids are shown separately, which shows that, the densities of SR molecules are spread deep into the bilayer. However, the Nitrogen atoms of LR lipids are at the same initial position similar to reference bilayer system. In Fig. 1h, we have shown the snapshot of 3.5 nm_AuNP system only with head group Nitrogen atoms of lipids. From partial densities of bilayer systems (see Fig. 2), it is evident that water is also penetrating along with the AuNP of different sizes. The water molecules and head group atoms of lipids interact with the AuNP because of the hydrophilic nature of nanoparticles. To quantify this, we have calculated distance distributions between lipid head group atoms (of interacting leaflet) and interacting surface gold atoms of AuNP and illustrated in Fig. 3. We have considered the gold atoms in each frame which are at the surface of the nanoparticle and in the vicinity of lipid molecules to calculate the distance distribution. These distributions are further normalized by number of interacting atoms of the lipid head groups. Distance distribution between head-group's non-ester phosphate Oxygen atoms (O4 and O5) and Gold atoms shows the higher peak height (3c) than other two atoms (Nitrogen and Carbon, Fig. 3a and b) which confirms the head-group's non-bridging (non-ester) Oxygen atoms are more ordered near to the AuNP surface. In Fig. S7, we have depicted separately the distance distribution between AuNP surface Gold atom and all phosphate Oxygen atoms (O3 and O6 for bridging, O4 and O5 double bonded) which show higher ordering of double bonded Oxygen atoms near AuNP surface. From Fig. 3a and b, it is evident that the Nitrogen and N-methyl Carbon atoms have smaller peak height, hence less ordering than Oxygen atoms. From Fig. 3, it is also clear that the size of the nanoparticle has no effect on the ordering of lipid atoms near the surface of AuNP. In Fig. 3d we have shown a snapshot of 3.5 nm_AuNP along with interacting lipid head group atoms for which we have plotted the distance distribution.

**Figure 4:**
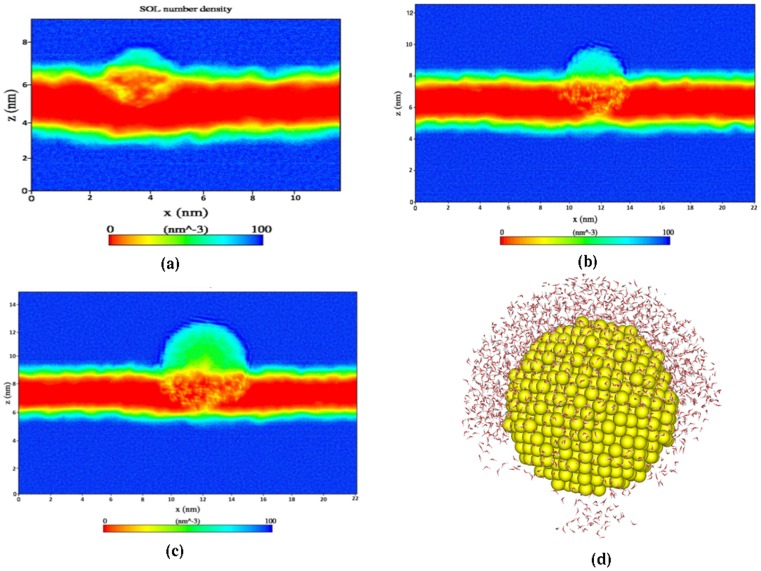
Two dimensional density map of water. (a), (b), and (c) represents systems 2 nm_AuNP, 3.5 nm_AuNP and 5 nm_AuNP respectively (d) schematically shows the water density around the AuNP upto certain cutoff (bilayer not shown).

As we have observed the water molecules also penetrated the bilayer along with AuNP, we have quantified the water density near to the AuNP. Two dimensional density map of water is calculated and presented in Fig. 4. From the densities of water near different sized nanoparticles we have observed that water penetrates into the lipid bilayer along with gold atoms. The density of water around the AuNP inside the bilayer is much less than the interfacial water density. It is also evident from the Fig. 4 that the water molecules, which enter the bilayer region along with AuNP remain discontinuous i.e., the water molecules are clustered near the AuNP surface. A snapshot representing this water cluster is shown in Fig. 4d. The two dimensional density calculated here is an average from last 10 ns of 100 ns trajectory. To our surprise the water clusters formed near the AuNP surface inside the bilayer region do not change its position and therefore shows discontinuity in the density profile. This provides a fact that, there is a clear competition between water oxygen and lipid head group atoms to interact with the hydrophilic AuNP. Therefore, only few water molecules penetrate the bilayer along with Gold atoms and get stabilized locally with the Gold surface atoms. Different sized gold nanoparticle has no effect in altering the local structure and the density of water around the AuNP (Fig. 4a, b and c).

### Comparison of structural and dynamical properties of short and long range lipids

It is obvious that the lipid molecules, which are interacting with AuNP will get affected the most and structural and dynamical properties of the lipid molecules will get altered. However the LR lipid molecules, which are much away from the adhesion site, also may get affected due to the indirect effect from the perturbation caused by AuNP. The different sizes of AuNP also may cause alteration in properties with different magnitude. Therefore, to understand the effect of nanoparticle, we have calculated structural and dynamical properties of the lipid molecules and compared with reference lipid system.

To understand the effect of perturbation of AuNP on lipid molecules, we have calculated separately the structural properties of head-groups and aliphatic tails of short (SR) and long range (LR) lipids independently and compared with the reference lipid bilayer system (without AuNP).

**Figure 5:**
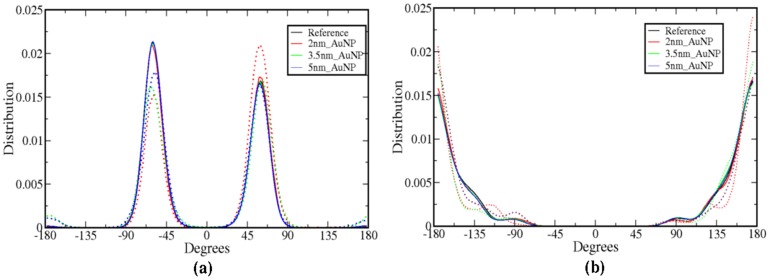
Dihedral angle distribution of the head-group atoms (a) N-C25-C24-O6 (b) C25-C24-O6-P. Dotted and solid lines are for SR and LR lipid molecules respectively.

The hydrophilic AuNP shows higher interaction with the hydrophilic atoms of the head-group of lipid molecules. Because of this interaction, the dihedrals between the head-group atoms can get altered. Therefore, we have calculated and compared the dihedral angle distribution of head-group atoms (N-C25-C24-O6 and C25-C24-O6-P) of SR and LR lipids (Fig. 5a, b). The deviation in the dihedral angle distribution is observed for SR molecules. C25-C24-O6-P dihedral (Fig. 5b) shows changes in peak position. This is may be because of higher interaction of Gold surface with the head group Oxygen atom, which is observed from the distance distributions (see Fig. 3). However, LR molecules show the same distribution as reference bilayer system. Similarly, we have calculated angle distributions between the head group atoms N-C25-C24, C25-C24-O6 and C24-O6-P from last 10 ns of 100 ns trajectory. There is not much difference in angles between adjacent atoms of head-group of SR and LR lipid molecules. The results are given in Fig. S8. The interacting atoms of SR lipids (with nanoparticle) like Nitrogen and Oxygen shows a slight deviation in angle with neighboring atoms compared to LR molecules. We do not observe significant differences in the angle between head-group atoms for different size of AuNPs. The predefined parameters for angle (force constant) for harmonic angle potentials result in large bonded interaction between atoms of angles, which does not get affected due to much weaker interaction caused by favorable van der Waals or electrostatics. Therefore, we do not see much of difference in angle of head-group atoms of SR lipid molecules.

**Figure 6:**
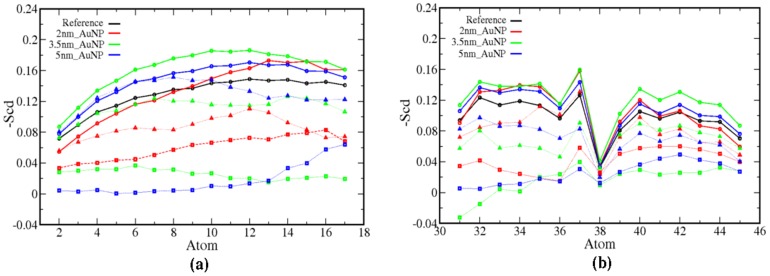
-S_cd_ order parameters of chains (a) *sn1* and (b) *sn2*. Symbols square, triangle and circle represents short range, buffer region and long range lipids.

**Figure 7:**
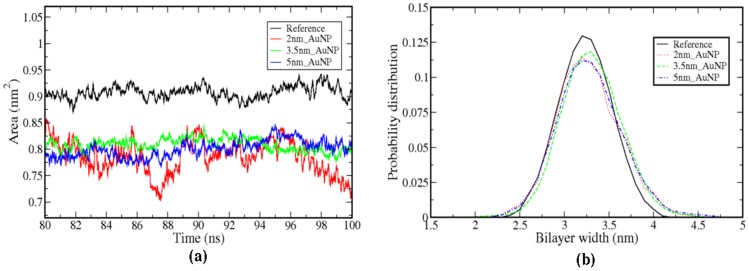
a) Area per head-group of LR lipids b) distribution of lipid bilayer thickness of the LR lipid region.

It is reported in the literature that a small perturbation to the bilayer can change the fluidity of the bilayer [18], [19], [39]. The fluidity of a bilayer is often related to the structural ordering of the lipid molecules and in this case it is mainly aliphatic tails of the lipids. In our simulations, we have used lipid molecules with one saturated (*sn1*) and other unsaturated (*sn2*) aliphatic chain (C37-C38 refer Fig. S1). To calculate the structural ordering of lipid tails we have calculated the order parameter -S_cd_ according to Chau et al. [40] and Tieleman et al. [41]. The order parameter is related to the tilt angle of the acyl chain with respect to the bilayer normal and gives indirect distribution of dihedral angles of the chain. So with the increase in the order parameter (-S_cd_) the trans conformation increase and tilt angle decrease. In Fig. 6a and b, we have illustrated the order parameter -S_cd_ for *sn1* and *sn2* chains of lipid as a function of carbon atom number (same number used as in Fig. S1). The order parameters are calculated for carbon atoms C17 to C2 of *sn1* chain and C31 to C45 of *sn2* chain (Fig. S1). From Fig. 6 it is evident that carbon atoms, which are located near to head-group (C17-C8) of *sn1*chain, are more ordered than *sn2* chain of LR lipid molecules. The carbon atom of end-group of tails of *sn1* and *sn2* chains shows less ordering because of their higher flexibility. Hence the bilayers, which are perturbed by different sizes of AuNP show more ordering of LR lipid tails than the reference bilayer. The lipids at buffer region are ordered compared to the SR region, however, not as ordered as LR region. The ordering of *sn1* and *sn2* tails of SR lipid molecules is much less than the LR lipids and reference bilayer system. So it is evident that the AuNP not only affects the lipid molecules in the vicinity, but also causes changes in structural properties of the lipid molecules far from the adhesion site. The observed enhancement of structural ordering of lipid tails can cause decrease in area per head group and increase the bilayer thickness. However, the effect can be seen in the region where LR lipid molecules are present. Therefore, we have computed the area per lipid of LR molecules. It is calculated by subtracting the area of SR molecules, including the buffer region from the total area of the box in bilayer plane. Thereafter, this area is divided by the number of lipid molecules present in the interacting leaflet in LR region and represented in Fig. 7a as a function of time (last 20 ns of 100 ns). We have observed for all the cases there is a slight decrease (6–7%) in area per head group of the LR lipid molecules. In Fig. S9, we have shown the transition of the area per head group as a function of time starting from 0 ns. This gives the direct evidence that the AuNP after getting inserted enough in the bilayer the area per head groups for the LR molecules get altered. The lipid order parameters, area per lipid and the bilayer thickness are the inter-related properties, as one can expect the enhanced lipid ordering can increase the bilayer thickness due to higher lipid packing. Therefore, to check this, we have calculated the bilayer thickness. It is carried out by dividing the simulation box in 50×50 grids using GridMAT-MD algorithm developed for bilayer analysis [42]. In each grid we have calculated the distances between nitrogen atoms of two leaflets and averaged it. Then the normalized distribution of the bilayer thickness is calculated from each grid for the LR lipid region and plotted in Fig. 7b. The distribution of bilayer thickness for the SR lipid region is given in Fig. S10. From Fig. 7b it is clear that the distributions of bilayer thickness shift towards higher value than the reference bilayer system. This is the direct evidence of the increase in packing of the LR lipid molecules because of a higher ordering of the aliphatic tails lipid molecules residing in the LR region.

**Figure 8:**
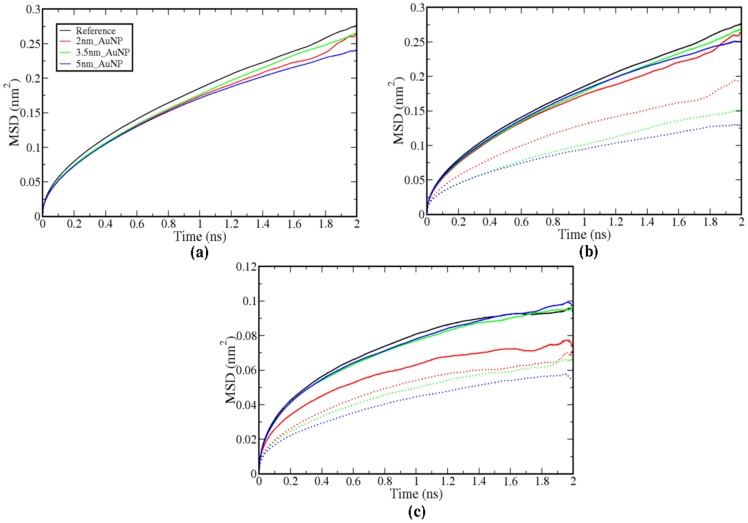
(a) Lateral MSD of all AOPC lipid in the system, (b) lateral MSD of (only interacting leaflet) and (c) MSD of interacting leaflet along bilayer normal. Dotted lines represent MSDs of SR lipids whereas solid lines are for LR lipid molecules.

The ordering of the lipid will directly affect the membrane fluidity. Therefore, we have checked the fluidity of the membrane from the mean square displacement (MSD) of SR and LR lipid molecules and compared with the reference bilayer lipid molecules. All the MSD's were obtained from last 20 ns and plotted as averaged MSD taken from all intervals of 2 ns each (Fig. S11). The lateral and normal to the bilayer MSDs are calculated and depicted in Fig. 8. In all the cases we have observed the decrease in MSDs due to the presence of the nanoparticle. The MSDs of SR molecules decreased because of direct interaction with AuNP. However, the decrease in lateral and normal to bilayer MSDs for LR molecules are even lesser than the SR molecules. Different sized nanoparticles show a similar decrease in MSDs, however the magnitudes are marginally same. The diffusion constants calculated from these MDS are given in Table 2. We have observed the decrease in the diffusion coefficient values for both SR and LR lipids. The lipid molecules much away from the AuNP interacting site indirectly get disturbed, which helps in the ordering the hydrophobic tails. This ordering enhances the packing of the hydrophobic part of the lipid molecules and thus reduces the fluidity of the molecules.

**Table 2:** Diffusion coefficient for SR and LR lipids.

System	SR Lipids (x10^7^ cm^2^/s)	LR Lipids (x10^7^ cm^2^/s)
**Reference**	–	1.50±0.145
**2 nm_AuNP**	0.98± 0.145	1.42± 0.101
**3.5 nm_AuNP**	0.86± 0.199	1.45± 0.176
**5 nm_AuNP**	0.62± 0.199	1.19±0.243

## Conclusion

We have demonstrated the effect of interaction of single AuNP on the lipid bilayer membrane by using all atomistic molecular dynamics simulations. AuNP can alter the structural properties of head-groups lipid molecules, which are directly interacting with the Gold atoms. We have observed local structural arrangements of lipid head-group near to the surface of the Gold atoms. The motivation of the work was to understand the effect the perturbation caused by the AuNP on the lipid molecules, which are away from the interacting site. To achieve this we have performed long simulations on sufficiently large bilayer AuNP systems. The results of these simulations are compared with the reference bilayer. The lipid molecules which are away from the AuNP adsorption site showed an enhancement in structural ordering of the hydrophobic tail. The higher ordering the bilayer thickness in the LR region turn out to be increased and area per head group got decreased. The packing of the lipid molecules in LR region became more, which results into relatively lesser fluidity of the LR lipid molecules. The different sizes AuNPs have the same effect on the bilayer. However, the magnitude of structural ordering and fluidity differs.

In this work we have dealt with only one AuNP at a time, so the nanoparticle concentration on the bilayer surface was very small. Marginally higher concentration of AuNP may enhance the effect of ordering to certain extent. However, much higher concentration will effectively increase deformation in the adsorption sites and can cause changes in bilayer properties. Therefore, understanding in molecular level the effect of higher the loading of NPs in the bilayer remains open for further investigation. In this work we have focused on the structural change of the lipid molecules in lesser time-scale. In larger time-scale NPs can alter the inter-leaflet motion of the lipid molecules, i.e., lipid flip-flop. Coarse-gain model of lipid and NP will be useful to address such dynamics of the lipid, which is beyond the scope of this paper.

## Supporting Information

Figure S1 Chemical structure of the AOPC lipid.
(TIFF)

Figure S2 Initial and final structures of AuNP (a,d) 2 nm_AuNP,(b,e) 3.5 nm_AuNPand (c,f) 5 nm_AuNP respectively.
(TIFF)

Figure S3 Distance distribution between Gold atoms in water and in vacuum. Black and red line shows Au-Au distance distribution in vacuum and water respectively.
(EPS)

Figure S4 (a) Distance distribution between Gold-Gold atoms and (b) RDF between Gold and Oxygen atoms of water. Colors red, green and blue represents 2 nm AuNP, 3.5 nm AuNP and 5 nm AuNP systems.
(TIFF)

Figure S5 Snapshots of AOPC lipid bilayer after equilibration run (a) 128 AOPC lipid system,(b) 512 AOPC lipid system
(TIFF)

Figure S6 The snapshots of the systems (a) 2 nm_AuNP, (b) 3.5 nm_AuNP and (c) 5 nm_AuNP as a function of time (0 ns, 25 ns, 50 ns, 75 ns and 100 ns).
(TIFF)

Figure S7 Distance distribution between AuNP surface Gold and Phosphate Oxygen atoms separately.
(EPS)

Figure S8 (a) Head-group atoms. Angle distribution of the head-group atoms (b) N-C25-C24 (c) C25-C24-O6 and (d) C24-O6-P.
(TIFF)

Figure S9 Area per head group for LR lipid molecules as function of time for 3.5 nm_AuNP system.
(EPS)

Figure S10 Distribution of lipid bilayer thickness of SR lipid region. Colors black, red, green and blue represents reference system, 2 nm AuNP, 3.5 nm AuNP and 5 nm AuNP systems respectively.
(EPS)

Figure S11 Lateral mean square displacement of AOPC lipids from last 20 ns with 2 ns interval of each. Black solid line represents average MSD of all intervals.
(EPS)

Table S1 Number of lipids in SR, buffer and LR region.
(DOCX)

Text S1 Forcefield: AOPC and AuNP.
(DOCX)
