# Supplementary figures and images for: Effect of Gold Nanoparticle on Structure and Fluidity of Lipid Membrane
Source: PLoS One. 2014 Dec 3;9(12):e114152. doi: 10.1371/journal.pone.0114152 (PMC4255040; doi:10.1371/journal.pone.0114152)

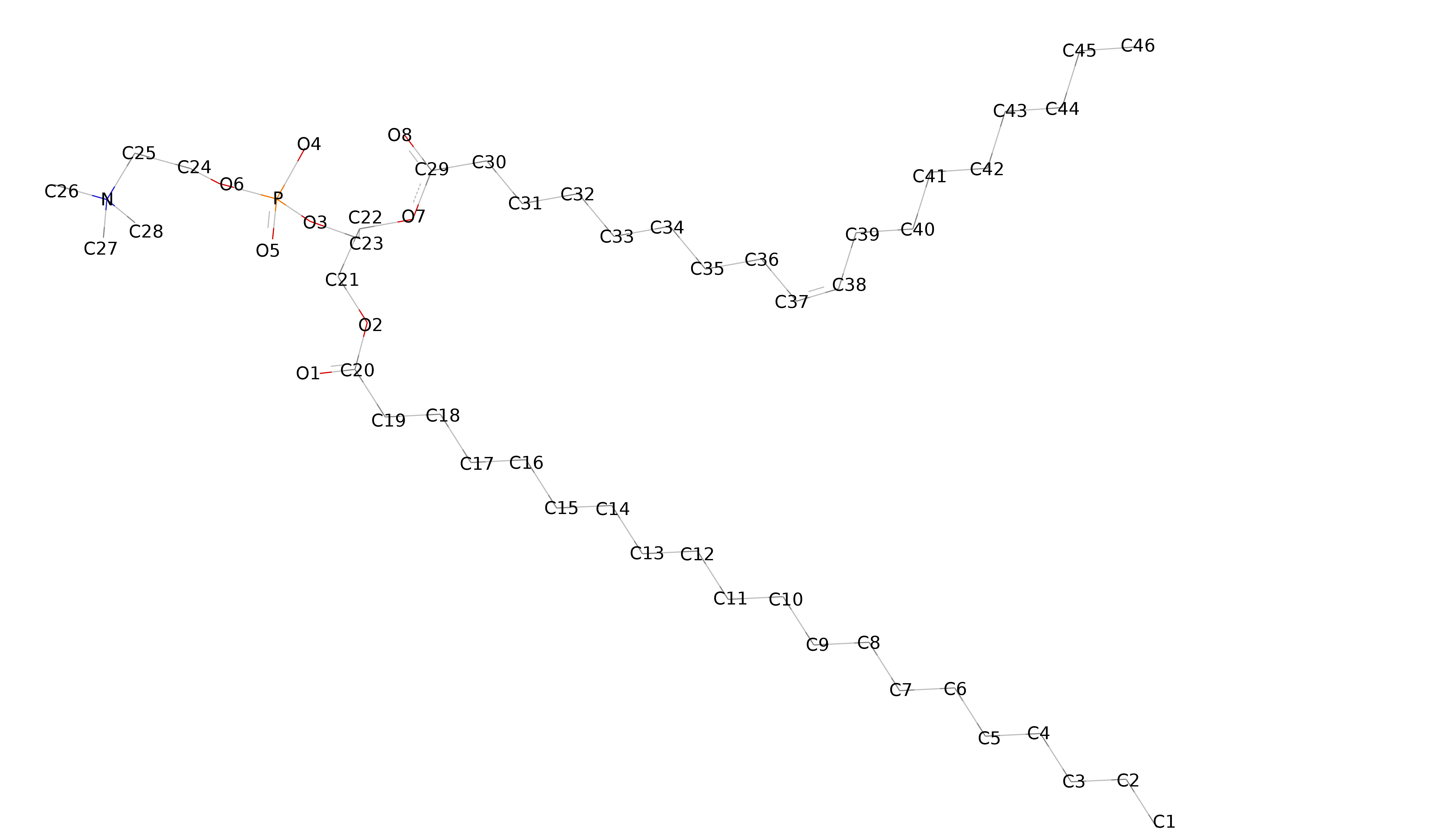

Supplement: Figure S1 — Chemical structure of the AOPC lipid. (TIFF) [file pone.0114152.s001.tiff]

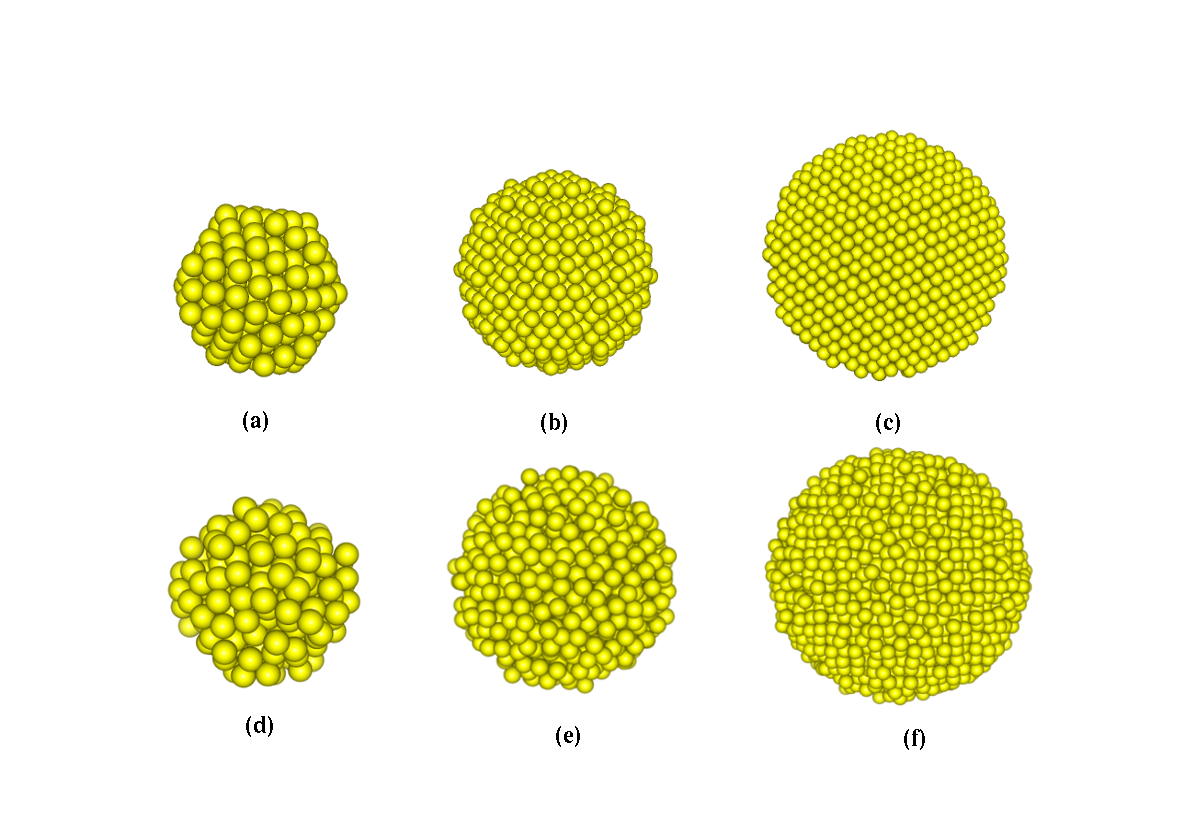

Supplement: Figure S2 — Initial and final structures of AuNP (a,d) 2 nm_AuNP,(b,e) 3.5 nm_AuNPand (c,f) 5 nm_AuNP respectively. (TIFF) [file pone.0114152.s002.tiff]

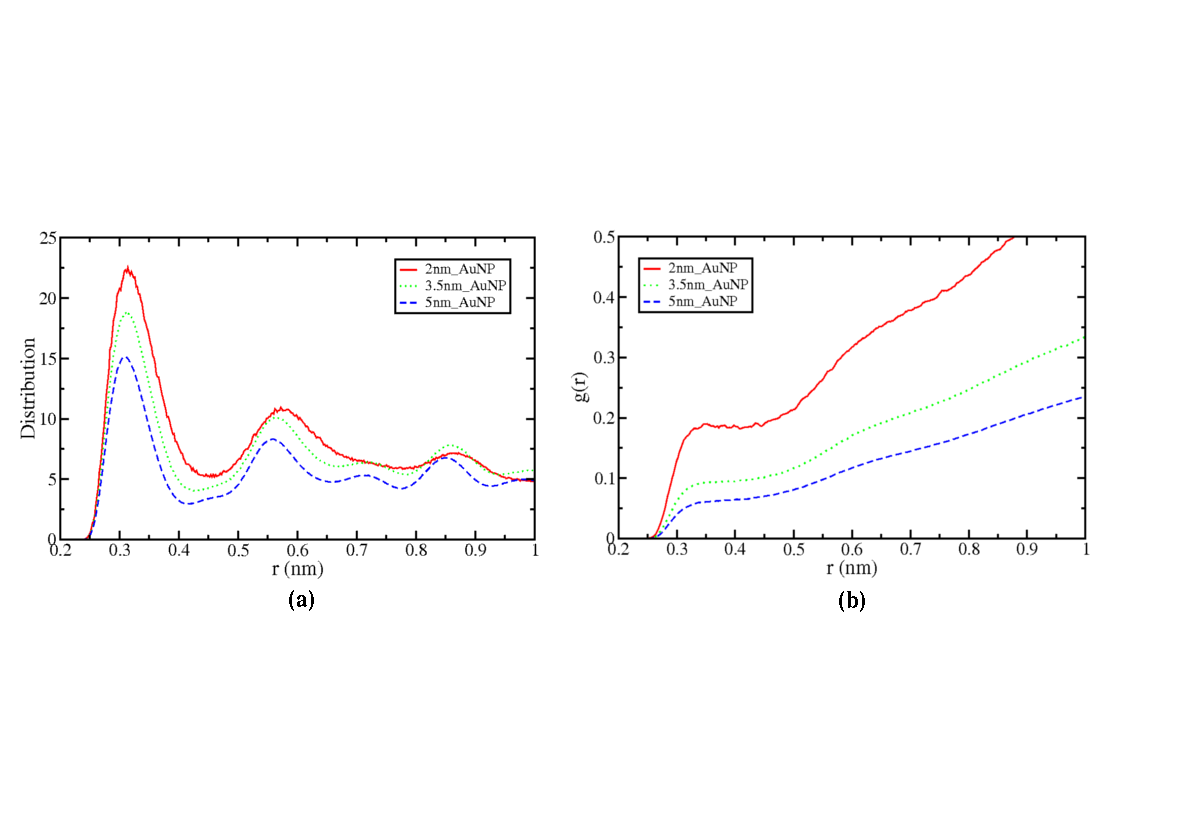

Supplement: Figure S4 — (a) Distance distribution between Gold-Gold atoms and (b) RDF between Gold and Oxygen atoms of water. Colors red, green and blue represents 2 nm AuNP, 3.5 nm AuNP and 5 nm AuNP systems. (TIFF) [file pone.0114152.s004.tiff]

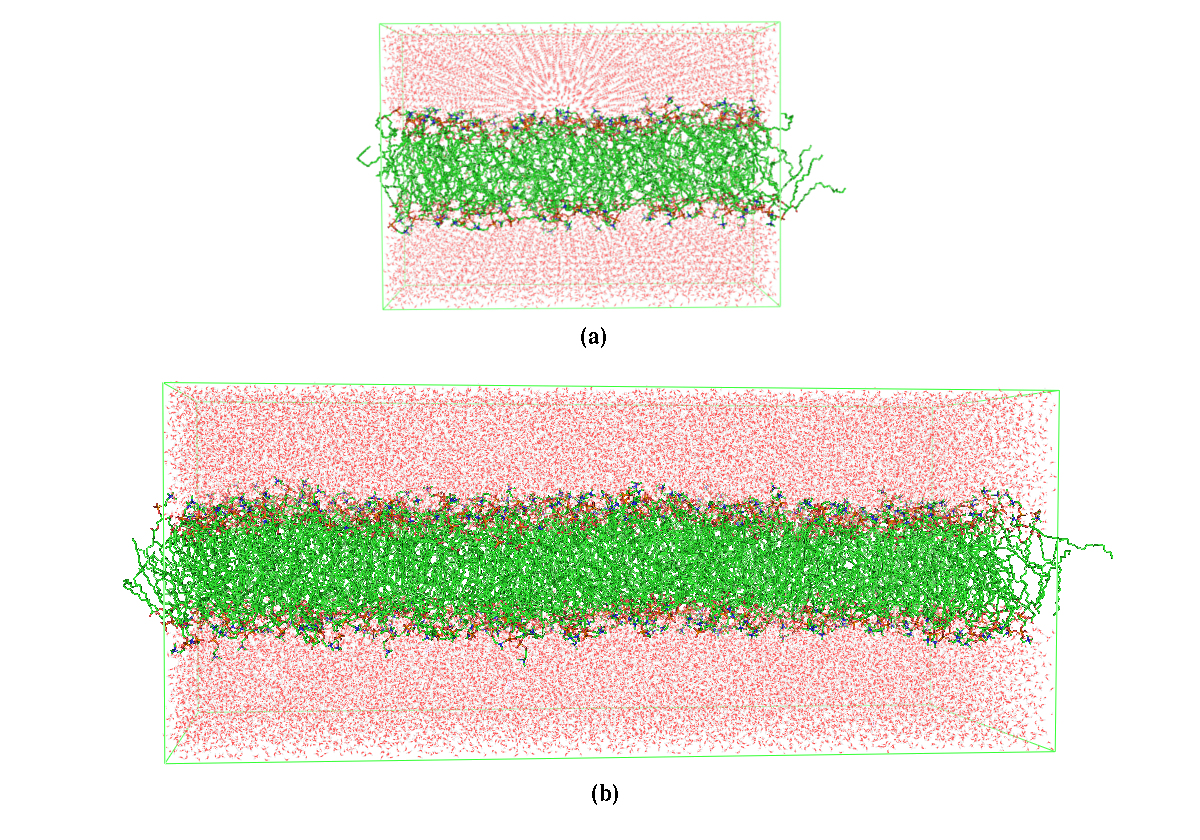

Supplement: Figure S5 — Snapshots of AOPC lipid bilayer after equilibration run (a) 128 AOPC lipid system,(b) 512 AOPC lipid system (TIFF) [file pone.0114152.s005.tiff]

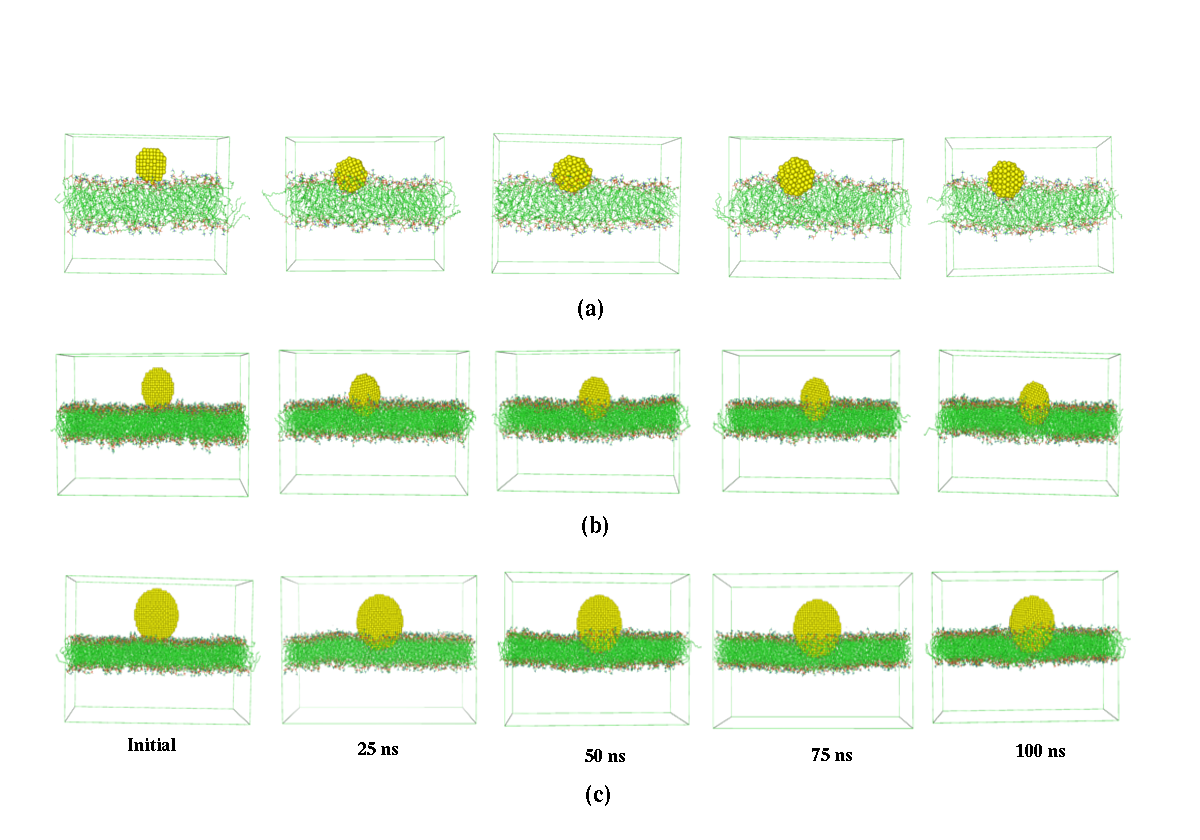

Supplement: Figure S6 — The snapshots of the systems (a) 2 nm_AuNP, (b) 3.5 nm_AuNP and (c) 5 nm_AuNP as a function of time (0 ns, 25 ns, 50 ns, 75 ns and 100 ns). (TIFF) [file pone.0114152.s006.tiff]

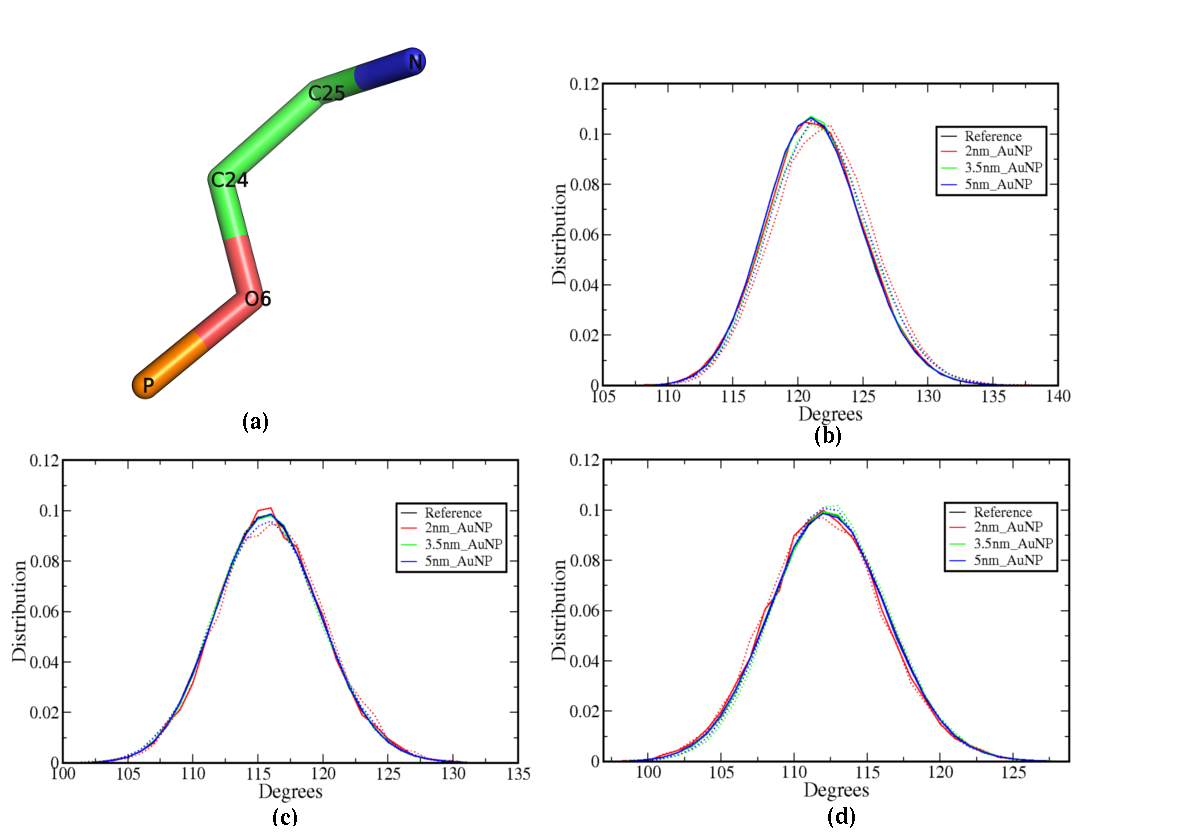

Supplement: Figure S8 — (a) Head-group atoms. Angle distribution of the head-group atoms (b) N-C25-C24 (c) C25-C24-O6 and (d) C24-O6-P. (TIFF) [file pone.0114152.s008.tiff]
