# Supplementary material for: Effect of Gold Nanoparticle on Structure and Fluidity of Lipid Membrane
Source: PLoS One. 2014 Dec 3;9(12):e114152. doi: 10.1371/journal.pone.0114152 (PMC4255040; doi:10.1371/journal.pone.0114152)
Supplement: Table S1 — Number of lipids in SR, buffer and LR region. (DOCX) [file pone.0114152.s012.docx]

Table S1. Number of lipids in SR, buffer and LR region.

| System | SR lipids | Buffer lipids | LR lipids |
| --- | --- | --- | --- |
| 2nm_AuNP | ~16 | ~10 | ~46 |
| 3.5nm_AuNP | ~44 | ~10 | ~218 |
| 5nm_AuNP | ~62 | ~10 | ~198 |
