# Supplementary material for: Effect of Gold Nanoparticle on Structure and Fluidity of Lipid Membrane
Source: PLoS One. 2014 Dec 3;9(12):e114152. doi: 10.1371/journal.pone.0114152 (PMC4255040; doi:10.1371/journal.pone.0114152)
Supplement: Text S1 — Forcefield: AOPC and AuNP. (DOCX) [file pone.0114152.s013.docx]

**Text S1**

**Forcefield : AOPC and AuNP**

[ moleculetype ]

; Name nrexcl

AOPC 3

[ atoms ]

; nr type resnr resid atom cgnr charge mass total_charge

1 CH3 1 AOPC C27 1 0.400 15.0350

2 CH3 1 AOPC C26 1 0.400 15.0350

3 CH3 1 AOPC C28 1 0.400 15.0350

4 NL 1 AOPC N 1 -0.500 14.0067

5 CH2 1 AOPC C25 1 0.300 14.0270 ; 1.000

6 CH2 1 AOPC C24 2 0.400 14.0270

7 OA 1 AOPC O6 2 -0.800 15.9994

8 P 1 AOPC P 2 1.700 30.9738

9 OM 1 AOPC O5 2 -0.800 15.9994

10 OM 1 AOPC O4 2 -0.800 15.9994

11 OA 1 AOPC O3 2 -0.700 15.9994 ; -1.000

12 CH2 1 AOPC C23 3 0.400 14.0270

13 CH1 1 AOPC C22 3 0.300 13.0190

14 OE 1 AOPC O7 3 -0.700 15.9994

15 CHO 1 AOPC C29 3 0.700 12.0110

16 O 1 AOPC O8 3 -0.700 15.9994 ; 0.000

17 CH2 1 AOPC C30 4 0.000 14.0270 ; 0.000

18 CH2 1 AOPC C31 5 0.000 14.0270 ; 0.000

19 CH2 1 AOPC C32 6 0.000 14.0270 ; 0.000

20 CH2 1 AOPC C33 7 0.000 14.0270 ; 0.000

21 CH2 1 AOPC C34 8 0.000 14.0270 ; 0.000

22 CH2 1 AOPC C35 9 0.000 14.0270 ; 0.000

23 CH2 1 AOPC C36 10 0.000 14.0270 ; 0.000

24 CR1 1 AOPC C37 11 0.000 13.0190 ; 0.000

25 CR1 1 AOPC C38 12 0.000 13.0190 ; 0.000

26 CH2 1 AOPC C39 13 0.000 14.0270 ; 0.000

27 CH2 1 AOPC C40 14 0.000 14.0270 ; 0.000

28 CH2 1 AOPC C41 15 0.000 14.0270 ; 0.000

29 CH2 1 AOPC C42 16 0.000 14.0270 ; 0.000

30 CH2 1 AOPC C43 17 0.000 14.0270 ; 0.000

31 CH2 1 AOPC C44 18 0.000 14.0270 ; 0.000

32 CH2 1 AOPC C45 19 0.000 14.0270 ; 0.000

33 CH3 1 AOPC C46 20 0.000 15.0350 ; 0.000

34 CH2 1 AOPC C21 21 0.500 14.0270

35 OE 1 AOPC O2 21 -0.700 15.9994

36 CHO 1 AOPC C20 21 0.800 12.0110

37 O 1 AOPC O1 21 -0.600 15.9994 ; 0.000

38 CH2 1 AOPC C19 22 0.000 14.0270 ; 0.000

39 CH2 1 AOPC C18 23 0.000 14.0270 ; 0.000

40 CH2 1 AOPC C17 24 0.000 14.0270 ; 0.000

41 CH2 1 AOPC C16 25 0.000 14.0270 ; 0.000

42 CH2 1 AOPC C15 26 0.000 14.0270 ; 0.000

43 CH2 1 AOPC C14 27 0.000 14.0270 ; 0.000

44 CH2 1 AOPC C13 28 0.000 14.0270 ; 0.000

45 CH2 1 AOPC C12 29 0.000 14.0270 ; 0.000

46 CH2 1 AOPC C11 30 0.000 14.0270 ; 0.000

47 CH2 1 AOPC C10 31 0.000 14.0270 ; 0.000

48 CH2 1 AOPC C9 32 0.000 14.0270 ; 0.000

49 CH2 1 AOPC C8 33 0.000 14.0270 ; 0.000

50 CH2 1 AOPC C7 34 0.000 14.0270 ; 0.000

51 CH2 1 AOPC C6 35 0.000 14.0270 ; 0.000

52 CH2 1 AOPC C5 36 0.000 14.0270 ; 0.000

53 CH2 1 AOPC C4 37 0.000 14.0270 ; 0.000

54 CH2 1 AOPC C3 38 0.000 14.0270 ; 0.000

55 CH2 1 AOPC C2 39 0.000 14.0270 ; 0.000

56 CH3 1 AOPC C1 40 0.000 15.0350 ; 0.000

; total charge of the molecule: 0.000

[ bonds ]

; ai aj funct c0 c1

1 4 2 0.1470 8.7100e+06

2 4 2 0.1470 8.7100e+06

3 4 2 0.1470 8.7100e+06

4 5 2 0.1470 8.7100e+06

5 6 2 0.1530 7.1500e+06

6 7 2 0.1430 8.1800e+06

7 8 2 0.1610 4.8400e+06

8 9 2 0.1480 8.6000e+06

8 10 2 0.1480 8.6000e+06

8 11 2 0.1610 4.8400e+06

11 12 2 0.1430 8.1800e+06

12 13 2 0.1530 7.1500e+06

13 14 2 0.1430 8.1800e+06

13 34 2 0.1530 7.1500e+06

14 15 2 0.1330 1.1800e+07

15 16 2 0.1230 1.6600e+07

15 17 2 0.1480 7.6400e+06

17 18 2 0.1530 7.1500e+06

18 19 2 0.1530 7.1500e+06

19 20 2 0.1530 7.1500e+06

20 21 2 0.1530 7.1500e+06

21 22 2 0.1530 7.1500e+06

22 23 2 0.1530 7.1500e+06

23 24 2 0.1530 7.1500e+06

24 25 2 0.1330 1.1800e+07

25 26 2 0.1530 7.1500e+06

26 27 2 0.1530 7.1500e+06

27 28 2 0.1530 7.1500e+06

28 29 2 0.1530 7.1500e+06

29 30 2 0.1530 7.1500e+06

30 31 2 0.1530 7.1500e+06

31 32 2 0.1530 7.1500e+06

32 33 2 0.1530 7.1500e+06

34 35 2 0.1430 8.1800e+06

35 36 2 0.1330 1.1800e+07

36 37 2 0.1230 1.6600e+07

36 38 2 0.1480 7.6400e+06

38 39 2 0.1530 7.1500e+06

39 40 2 0.1530 7.1500e+06

40 41 2 0.1530 7.1500e+06

41 42 2 0.1530 7.1500e+06

42 43 2 0.1530 7.1500e+06

43 44 2 0.1530 7.1500e+06

44 45 2 0.1530 7.1500e+06

45 46 2 0.1530 7.1500e+06

46 47 2 0.1530 7.1500e+06

47 48 2 0.1530 7.1500e+06

48 49 2 0.1530 7.1500e+06

49 50 2 0.1530 7.1500e+06

50 51 2 0.1530 7.1500e+06

51 52 2 0.1530 7.1500e+06

52 53 2 0.1530 7.1500e+06

53 54 2 0.1530 7.1500e+06

54 55 2 0.1530 7.1500e+06

55 56 2 0.1530 7.1500e+06

[ pairs ]

; ai aj funct ; all 1-4 pairs but the ones excluded in GROMOS itp

1 6 1

2 6 1

3 6 1

4 7 1

5 8 1

6 9 1

6 10 1

6 11 1

7 12 1

8 13 1

9 12 1

10 12 1

11 14 1

11 34 1

12 15 1

12 35 1

13 16 1

13 17 1

13 36 1

14 18 1

14 35 1

15 19 1

15 34 1

16 18 1

17 20 1

18 21 1

19 22 1

20 23 1

21 24 1

22 25 1

24 27 1

25 28 1

26 29 1

27 30 1

28 31 1

29 32 1

30 33 1

34 37 1

34 38 1

35 39 1

36 40 1

37 39 1

38 41 1

39 42 1

40 43 1

41 44 1

42 45 1

43 46 1

44 47 1

45 48 1

46 49 1

47 50 1

48 51 1

49 52 1

50 53 1

51 54 1

52 55 1

53 56 1

[ angles ]

; ai aj ak funct angle fc

1 4 2 2 109.50 520.00

1 4 3 2 109.50 520.00

1 4 5 2 109.50 520.00

2 4 3 2 109.50 520.00

2 4 5 2 109.50 520.00

3 4 5 2 109.50 520.00

4 5 6 2 111.00 530.00

5 6 7 2 111.00 530.00

6 7 8 2 120.00 530.00

7 8 9 2 109.60 450.00

7 8 10 2 109.60 450.00

7 8 11 2 103.00 420.00

8 11 12 2 120.00 530.00

9 8 10 2 120.00 780.00

9 8 11 2 109.60 450.00

10 8 11 2 109.60 450.00

11 12 13 2 111.00 530.00

12 13 14 2 109.50 520.00

12 13 34 2 109.50 520.00

13 14 15 2 117.00 635.00

13 34 35 2 111.00 530.00

14 13 34 2 109.50 520.00

14 15 16 2 122.00 700.00

14 15 17 2 113.00 545.00

15 17 18 2 111.00 530.00

16 15 17 2 125.00 750.00

17 18 19 2 111.00 530.00

18 19 20 2 111.00 530.00

19 20 21 2 111.00 530.00

20 21 22 2 111.00 530.00

21 22 23 2 111.00 530.00

22 23 24 2 111.00 530.00

23 24 25 2 120.00 560.00

24 25 26 2 120.00 560.00

25 26 27 2 111.00 530.00

26 27 28 2 111.00 530.00

27 28 29 2 111.00 530.00

28 29 30 2 111.00 530.00

29 30 31 2 111.00 530.00

30 31 32 2 111.00 530.00

31 32 33 2 111.00 530.00

34 35 36 2 117.00 635.00

35 36 37 2 122.00 700.00

35 36 38 2 113.00 545.00

36 38 39 2 111.00 530.00

37 36 38 2 125.00 750.00

38 39 40 2 111.00 530.00

39 40 41 2 111.00 530.00

40 41 42 2 111.00 530.00

41 42 43 2 111.00 530.00

42 43 44 2 111.00 530.00

43 44 45 2 111.00 530.00

44 45 46 2 111.00 530.00

45 46 47 2 111.00 530.00

46 47 48 2 111.00 530.00

47 48 49 2 111.00 530.00

48 49 50 2 111.00 530.00

49 50 51 2 111.00 530.00

50 51 52 2 111.00 530.00

51 52 53 2 111.00 530.00

52 53 54 2 111.00 530.00

53 54 55 2 111.00 530.00

54 55 56 2 111.00 530.00

[ dihedrals ]

; GROMOS improper dihedrals

; ai aj ak al funct angle fc

13 14 34 12 2 35.26 334.72

15 14 16 17 2 0.00 167.36

23 24 25 26 2 0.00 167.36

36 35 37 38 2 0.00 167.36

[ dihedrals ]

; ai aj ak al funct ph0 cp mult

1 4 5 6 1 0.00 3.77 3

2 4 5 6 1 0.00 3.77 3

3 4 5 6 1 0.00 3.77 3

4 5 6 7 1 180.00 5.86 1

4 5 6 7 1 0.00 8.62 3

5 6 7 8 1 0.00 3.77 3

6 7 8 11 1 0.00 5.09 2

6 7 8 11 1 0.00 3.19 3

7 8 11 12 1 0.00 5.09 2

7 8 11 12 1 0.00 3.19 3

8 11 12 13 1 0.00 3.77 3

11 12 13 34 1 0.00 5.92 3

12 13 14 15 1 0.00 3.77 3

12 13 34 35 1 0.00 5.92 3

13 14 15 17 1 180.00 24.00 2

13 34 35 36 1 0.00 3.77 3

14 15 17 18 1 0.00 1.00 6

15 17 18 19 1 0.00 5.92 3

17 18 19 20 1 0.00 5.92 3

18 19 20 21 1 0.00 5.92 3

19 20 21 22 1 0.00 5.92 3

20 21 22 23 1 0.00 5.92 3

21 22 23 24 1 0.00 5.92 3

22 23 24 25 1 0.00 1.00 6

24 25 26 27 1 0.00 1.00 6

25 26 27 28 1 0.00 5.92 3

26 27 28 29 1 0.00 5.92 3

27 28 29 30 1 0.00 5.92 3

28 29 30 31 1 0.00 5.92 3

29 30 31 32 1 0.00 5.92 3

30 31 32 33 1 0.00 5.92 3

34 35 36 38 1 180.00 24.00 2

35 36 38 39 1 0.00 1.00 6

36 38 39 40 1 0.00 5.92 3

38 39 40 41 1 0.00 5.92 3

39 40 41 42 1 0.00 5.92 3

40 41 42 43 1 0.00 5.92 3

41 42 43 44 1 0.00 5.92 3

42 43 44 45 1 0.00 5.92 3

43 44 45 46 1 0.00 5.92 3

44 45 46 47 1 0.00 5.92 3

45 46 47 48 1 0.00 5.92 3

46 47 48 49 1 0.00 5.92 3

47 48 49 50 1 0.00 5.92 3

48 49 50 51 1 0.00 5.92 3

49 50 51 52 1 0.00 5.92 3

50 51 52 53 1 0.00 5.92 3

51 52 53 54 1 0.00 5.92 3

52 53 54 55 1 0.00 5.92 3

53 54 55 56 1 0.00 5.92 3

[ exclusions ]

; ai aj funct ; GROMOS 1-4 exclusions

23 26

**AuNP non-bonded parameters**

[ atomtypes ]

;name at.num mass charge ptype c6 c12

Au 79 0.000 0.000 A 0.0584122 3.85761e-05
